# Measuring AI literacy in medical students: scale development and validation within a self-determination theory framework

**DOI:** 10.1080/10872981.2026.2675066

**Published:** 2026-05-22

**Authors:** Hung-Che Lin, Chin-Sheng Lin, Ching Sing Chai, Chin Lin, Pei-Jan Tsai, Jyh-Chong Liang

**Affiliations:** a Department of Otolaryngology-Head and Neck Surgery, Tri-Service General Hospital, National Defense Medical University, Taipei, Taiwan; Department of Otolaryngology, School of Medicine, College of Medicine, National Defense Medical University, Taipei, Taiwan; b Department of Medical Education, Tri-Service General Hospital, National Defense Medical University, Taipei, Taiwan; c Division of Cardiology, Department of Internal Medicine, Tri-Service General Hospital, National Defense Medical University, Taipei, Taiwan; d School of Medicine, College of Medicine, National Defense Medical University, Taipei, Taiwan; e Department of Curriculum and Instruction, Faculty of Education, The Chinese University of Hong Kong, Hong Kong; f Program of Learning Sciences and Institute for Research Excellence in Learning Sciences, National Taiwan Normal University, Taipei, Taiwan

**Keywords:** Artificial intelligence, literacy, medical education, self-determination theory (SDT), structural equation modeling (SEM)

## Abstract

**Background:**

Artificial intelligence (AI) is increasingly integrated into healthcare, making AI literacy an essential competency for medical students. Existing assessments are often generic, lack validation in medical education, and are not grounded in learning theory. This study developed and validated the AI Literacy Scale for Medical Students (ALSMS) within a self-determination theory (SDT) framework.

**Methods:**

We used a split-sample validation design (*N* = 518; exploratory factor analysis [EFA], *n* = 204; confirmatory factor analysis [CFA], *n* = 314). Candidate facets were derived from prior AI literacy instruments and a previously developed framework, then organized according to SDT. EFA refined the first-order structure, and CFA cross-validated the retained structure and compared prespecified first-order and SDT-aligned higher-order models.

**Results:**

EFA identified nine factors organized into the SDT domains of competence, relatedness, and autonomy. CFA supported the correlated nine-factor structure and demonstrated strong psychometric properties. Model comparisons identified two theory-consistent, well-fitting solutions: a correlated nine-factor model and an SDT-aligned second-order model with *Ethics* loading on *Autonomy*. Unidimensional and some hierarchical general-factor models showed poorer fit or identification problems, supporting the construct’s multidimensionality.

**Conclusions:**

This study provides initial validity evidence for interpreting ALSMS scores as indicators of medical students’ AI literacy within an SDT-informed framework. The findings highlight the significance of integrating ethics into autonomy-supportive curricula and underscore the potential utility of ALSMS for curriculum design, advising, and the evaluation of AI literacy initiatives in medical education.

## Introduction

Artificial intelligence (AI) is rapidly reshaping healthcare, improving diagnostic accuracy, accelerating drug discovery, and supporting patient-centred care, while enabling the analysis of large-scale clinical data [1–5]. Alongside these opportunities, the deployment of AI raises significant ethical and governance concerns, such as algorithmic bias, privacy, accountability, and the need for appropriate human oversight, which must be addressed in medical training [6–10].

Against this background, we define ‘medical AI’ as the range of AI-enabled tools and workflows that medical learners increasingly encounter in healthcare settings (e.g., medical image analysis systems, AI-enabled voice consultation tools, and AI-supported clinical decision-support applications) [1], and we anchor instrument development in Self-Determination Theory (SDT), which posits that *Competence*, *Relatedness*, and *Autonomy* underlie motivated learning and professional growth [11–14]. Framing AI literacy within SDT enables simultaneous attention to knowledge and technical competencies, social and contextual supports, and value-aligned, ethical engagement with these technologies in clinical settings [15].

Medical education has begun to respond, yet implementation remains heterogeneous across institutions and programmes. A coherent pedagogical focus on AI literacy is needed. We adopt the widely cited definition of AI literacy by Long and Magerko, which frames it as a set of competencies that enable individuals to critically evaluate AI technologies, communicate and collaborate with AI, and utilise AI tools across various contexts [16]. Building on reviews of AI literacy in education, we argue that this concept requires contextualisation for medical learners, who face domain-specific clinical, ethical, and interprofessional demands [15,17–19].

Despite growing interest, measurement in this area lags behind. Existing instruments in medical education typically assess AI readiness, attitudes, or perceptions rather than AI literacy itself [20]. Recent studies have also relied on pre-validated but non-specialised tools [21,22], limiting their ability to capture the clinical, ethical, and interprofessional demands faced by medical learners.

Recent scale-development studies have advanced the broader field of AI-related measurement. Nong et al. (2024) developed and validated an AI literacy scale comprising application ability, cognitive ability, morality, critical thinking, and self-efficacy, providing an important multidimensional starting point for AI literacy assessment [23]. However, their instrument was developed for general AI literacy rather than the medical-education context, where learners must engage with AI amid clinical responsibility, ethical governance, and human oversight. Demirci et al. (2025) further demonstrated the value of theory-driven scale development by developing and validating an SDT-based motivation scale for generative AI-based language learning [24]. Although their study supports the suitability of SDT for AI-related educational measurement, it focused on motivation in language learning rather than AI literacy in medical education. These studies show that AI-related constructs benefit from multidimensional and theoretically grounded measurement, while also underscoring the need for a medical-education-specific, SDT-informed instrument to assess AI literacy.

To address these limitations, this study developed and validated the AI Literacy Scale for Medical Students (ALSMS) within a Self-Determination Theory framework. Specifically, we aimed to (1) construct an SDT-informed, multidimensional measure of AI literacy tailored to medical education; (2) examine its internal structure and reliability through exploratory and confirmatory analyses; and (3) evaluate whether SDT-aligned higher-order representations provide a parsimonious summary of the retained facets.

### Theoretical framework and literature review

#### Self-determination theory (SDT): rationale and construct mapping

Building on the rationale for an SDT-anchored approach introduced in the previous section, this section specifies how SDT informs the construct space of the **AI Literacy Scale for Medical Students (ALSMS)** and how these constructs were operationalized for measurement. Rather than revisiting general motivational claims, we focus on mapping SDT needs to hypothesised facets tailored to medical AI learning [12,13].

#### Why SDT for ALSMS?

SDT identifies three basic psychological needs, *Competence*, *Relatedness*, and *Autonomy*, whose satisfaction supports effective and sustained learning [11–13,25]. Given SDT’s extensive empirical support in education, including meta-analytic evidence linking need-supportive contexts to more favourable motivational and learning outcomes, we selected SDT as a well-validated motivational lens for organising facets of AI literacy [12,25,26]. This rationale is also consistent with recent AI-related scale-development work showing that SDT can serve as a coherent framework for organising learner-related constructs and that AI-related measures still require contextual adaptation to the specific professional and ethical demands of medical education [23,24]. Within SDT, contexts that support (rather than thwart) these needs promote need satisfaction and internalisation, facilitating a shift from more controlled forms of motivation toward more autonomous (self-endorsed) regulation, conditions associated with greater engagement, persistence, and learner well-being [11–13,25]. In developing ALSMS, we adopted a facets-first strategy: we first assembled ten candidate facets from prior AI-literacy instruments [16–19] and the existing AI learning intention framework and related prior work [3,27], and then mapped these facets onto SDT’s three needs to provide a theory-informed organising lens. We subsequently used split-sample EFA and CFA to examine the internal structure of the facets and conducted theory-driven CFA model comparisons to evaluate the extent to which SDT-aligned higher-order representations offer a parsimonious account of the relations among these facets (see Methods; Models a-g).

#### Competence domain: knowledge, skills, and efficacy for engaging with AI

Within SDT, *Competence* refers to the felt effectiveness in mastering tasks and achieving desired outcomes. We index competence with three facets relevant to medical AI:

1. *Basic knowledge*: conceptual and technical understanding of how AI functions and is applied in healthcare, which predicts intentions to engage with AI in learning contexts [3,27,28].
2. *Self-efficacy in AI learning*: confidence to learn with and about AI that supports engagement and persistence [25,27,29].
3. *Algorithms*: awareness of algorithmic functioning and limits in clinical decision support, including attention to bias and potential downstream harms if misused [7,9,18,30].

#### Relatedness domain: social-contextual supports for AI learning

*Relatedness* refers to the feeling of being connected and supported by others. In medical education, social, contextual, and patient-oriented supports foster participation in complex AI-related learning. We capture relatedness with:

1. *Innovative design for medical benefits*: students’ orientation toward improving medical AI applications for patient benefit, patient populations, and patient care, which links AI learning to the welfare of others and to shared clinical goals rather than to individual technical skill alone [31].
2. *Access to support and technology*: the availability of information, open-source resources, and help when students need to learn more about medical AI, which creates a connected learning environment that supports participation in AI-related learning [32].
3. *Supportive social norms*: encouragement and endorsement from parents, teachers, mentors, and classmates, which indicate that learning about medical AI is valued by important others and by the surrounding educational community [3,33].

#### Autonomy domain: value-aligned, self-directed engagement with AI

*Autonomy* reflects volitional, value-congruent regulation of learning and practice. Autonomy-supportive instruction is associated with deeper internalisation and improved outcomes. We operationalise autonomy with four facets:

1. *Personal relevance*: perceiving AI technologies as personally meaningful and useful for one’s own clinical learning, including task completion, performance, productivity, and efficiency, which supports internalisation and autonomous regulation [34,35].
2. *Ethics*: reasoned positions on trustworthy AI (e.g., oversight, privacy, fairness, accountability) and professional responsibility in clinical use. While ethics could theoretically align with relatedness (professional norms) or competence (governance knowledge), we **a priori** locate it under *Autonomy* as a value commitment shaping self-endorsed decisions about AI in care [6,17,18,36].
3. *Optimism*: a positive expectancy toward medical AI and a willingness to engage constructively with AI-related change under uncertainty [37]. In our operationalization, optimism reflects a self-directed orientation toward future learning and adaptation, which we treat as autonomy-relevant volitional regulation.
4. *Resilience*: adaptive persistence and constructive self-development in response to the growing role of medical AI [38–40]. Autonomy-supportive instruction has been linked to learners’ resilient functioning through increased agentic engagement and internalisation processes [38]. At the same time, we recognise that optimism and resilience are also closely related to efficacy beliefs and may be reasonably conceptualised as competence-adjacent psychological resources. Consistent with our facets-first approach, we therefore treat their placement under *Autonomy* as theoretically defensible but not exclusive, and we examine an alternative higher-order placement in a sensitivity model (see Methods; Model comparison).

#### Summary and link to the measurement models

**Figure 1.**
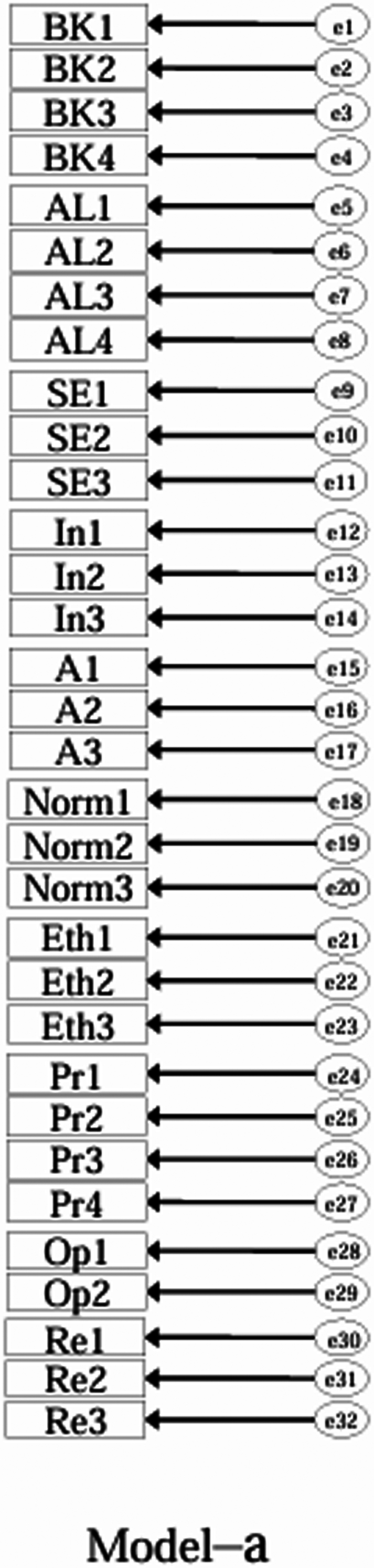
**Model-a:** Independence (null) model, in which all observed indicators are assumed to be uncorrelated.

**Figure 2.**
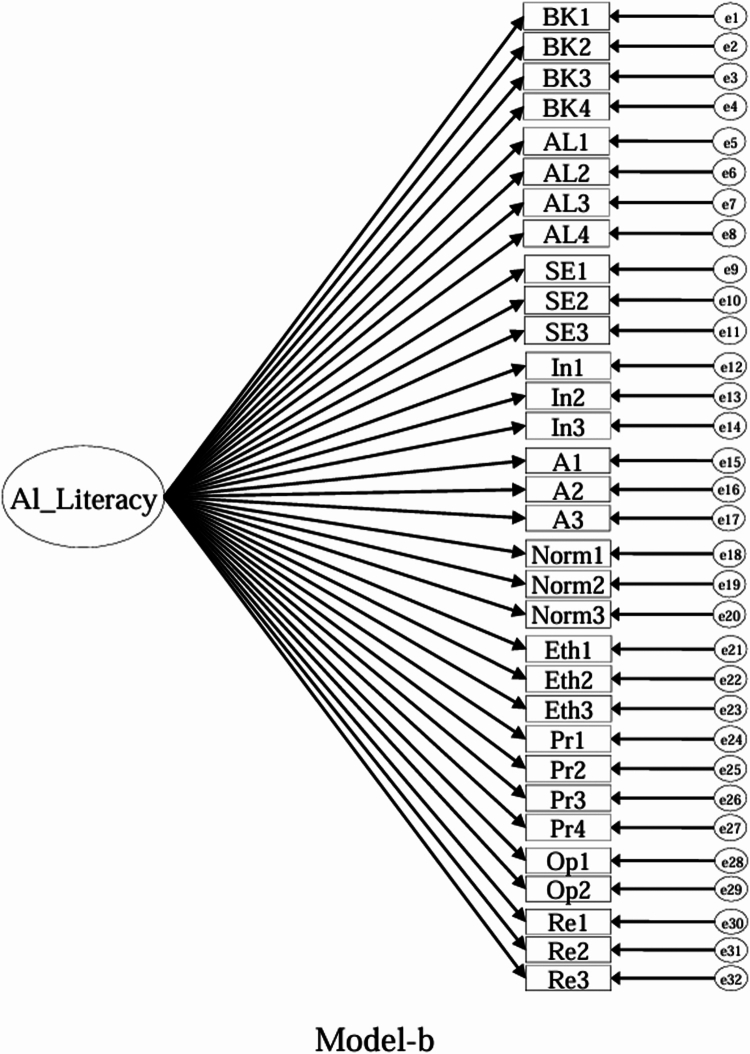
**Model-b:** First-order one-factor model, in which all ALSMS items load on a single latent AI-literacy factor.

**Figure 3.**
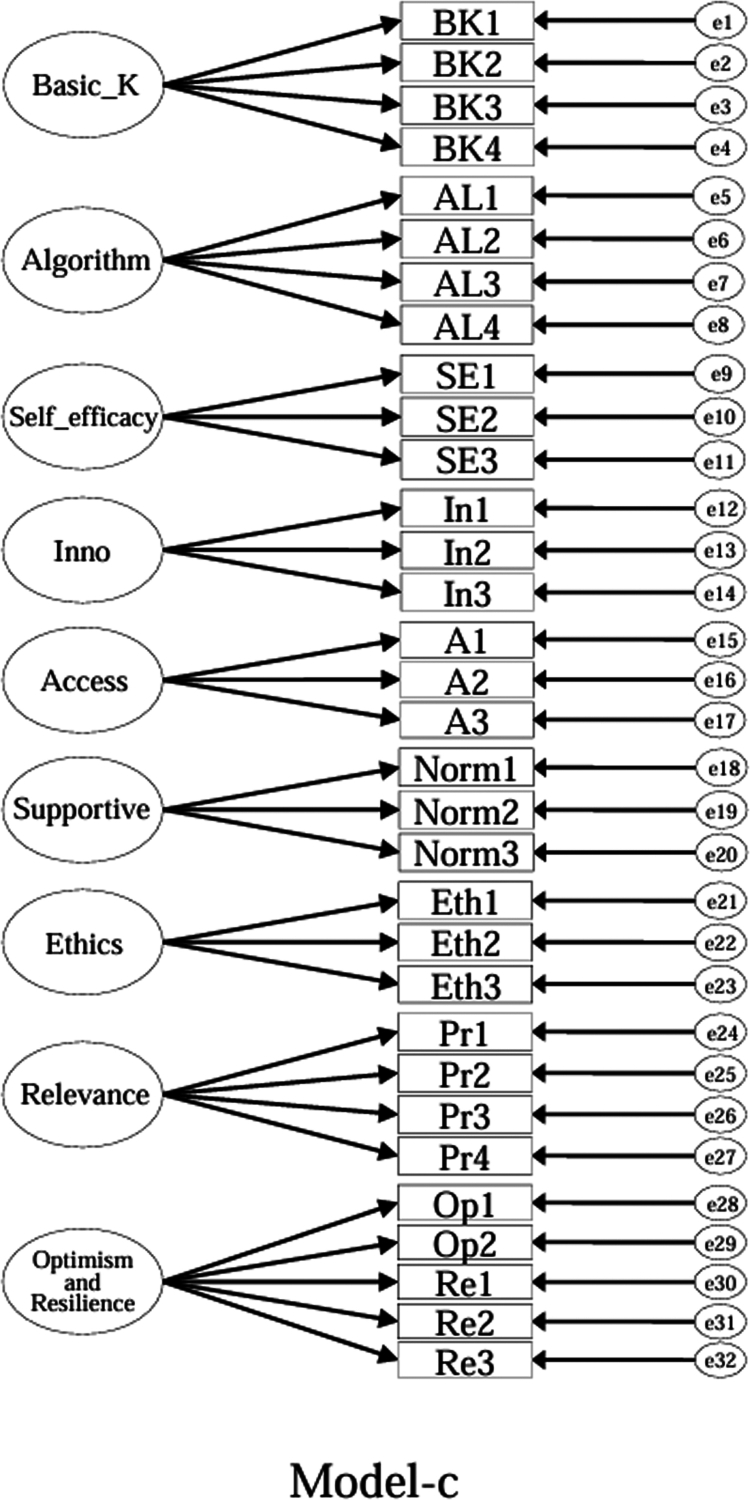
**Model-c:** First-order uncorrelated model, in which the nine facet factors are constrained to have zero correlations.

**Figure 4.**
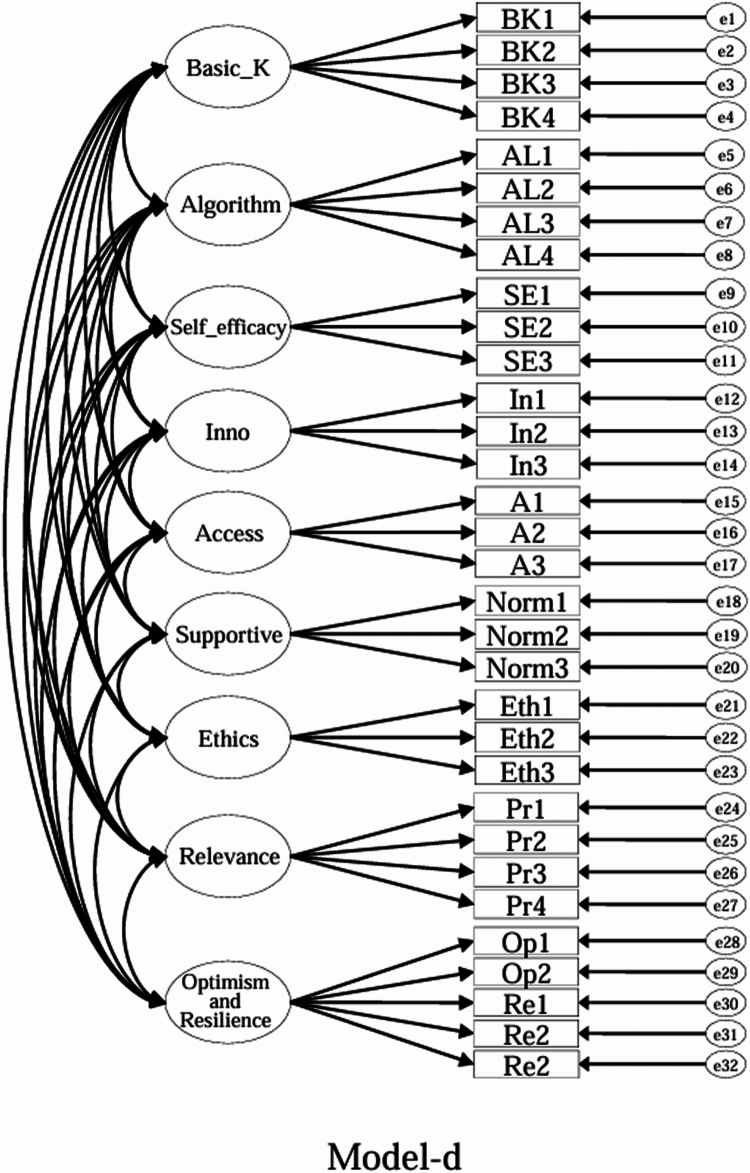
**Model-d:** First-order **correlated** model, in which the nine facet factors are freely intercorrelated.

**Figure 5.**
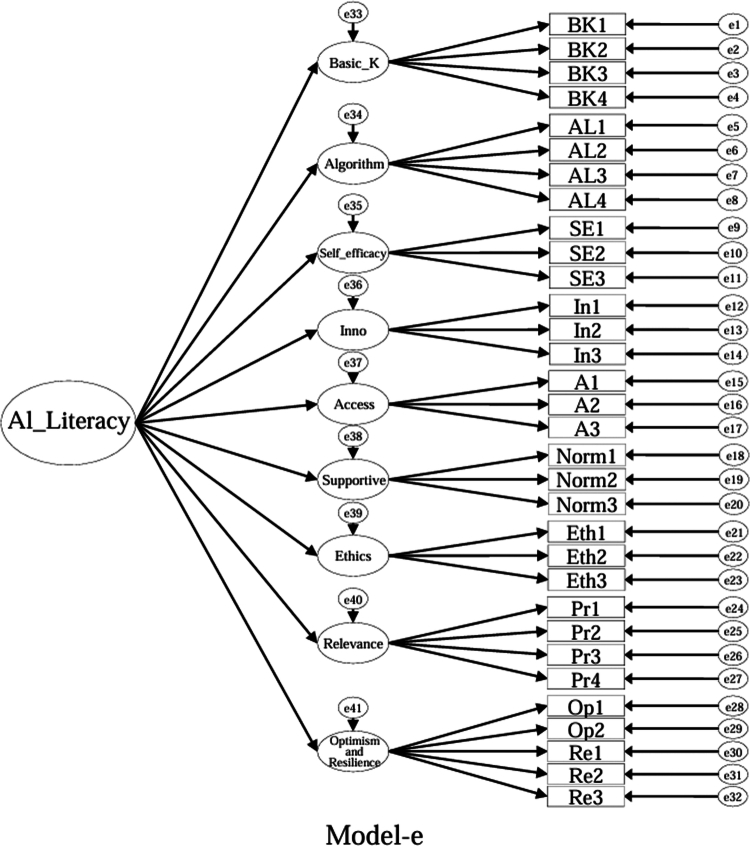
**Model-e:** Second-order general-factor model, in which the nine first-order facet factors load on a single higher-order AI-literacy factor.

**Figure 6.**
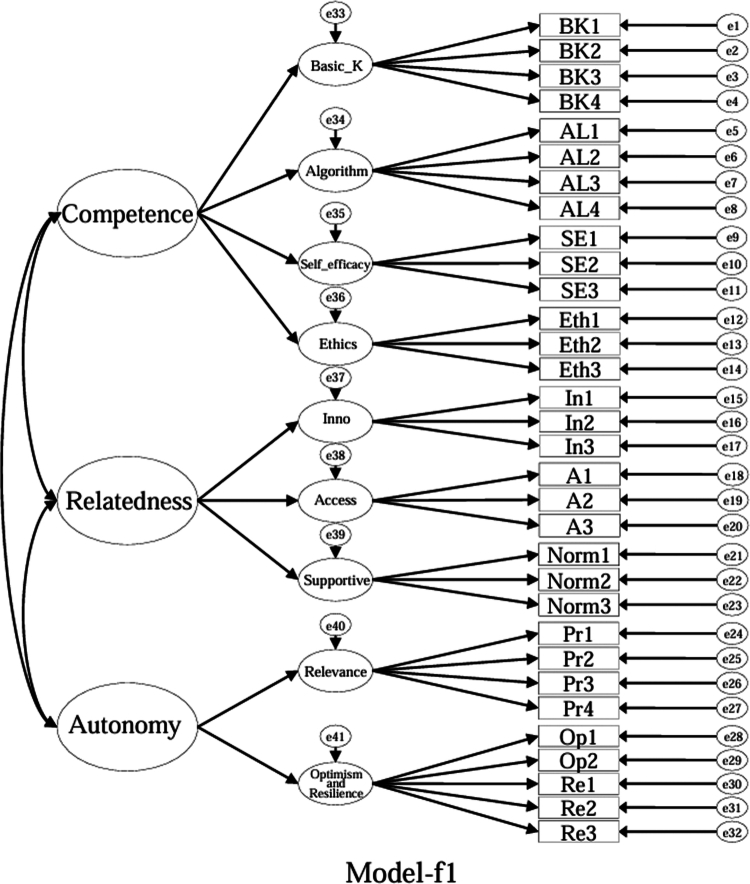
**Model-f1**: SDT-aligned second-order model with *Ethics* specified under *Relatedness* (inadmissible solution).

**Figure 7.**
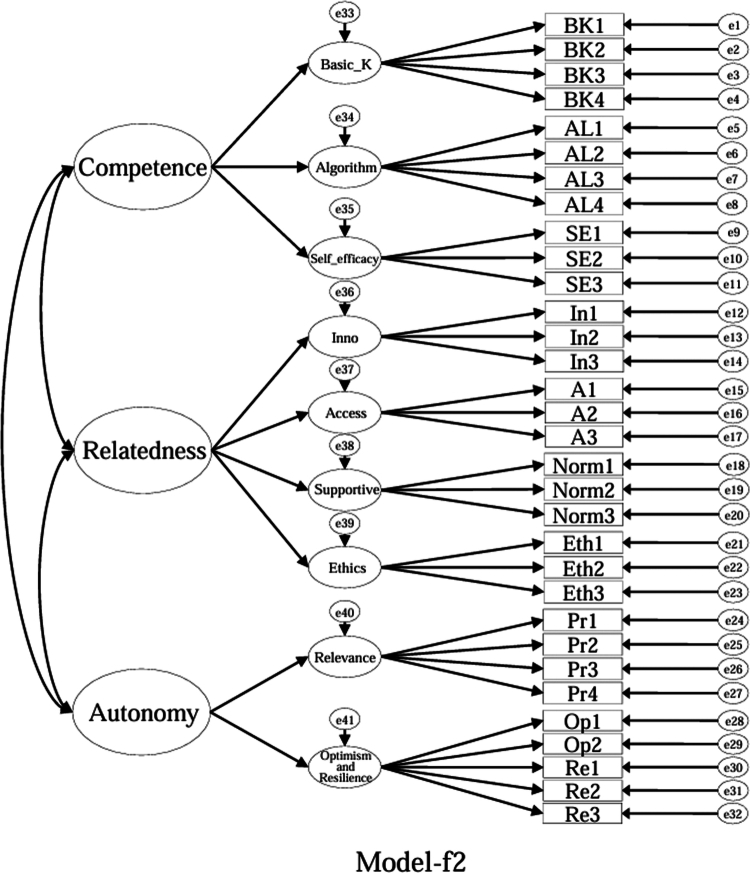
**Model-f2**: SDT-aligned second-order model with *Ethics* specified under *Competence* (inadmissible solution).

**Figure 8.**
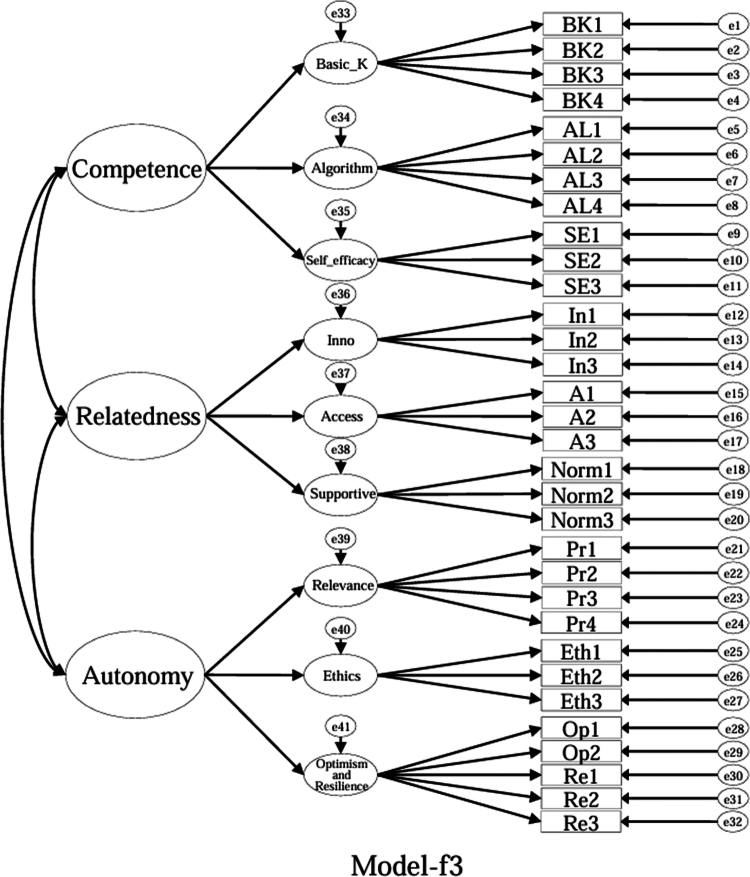
**Model-f3**: SDT-aligned second-order model with *Ethics* specified under *Autonomy* (admissible solution).

**Figure 9.**
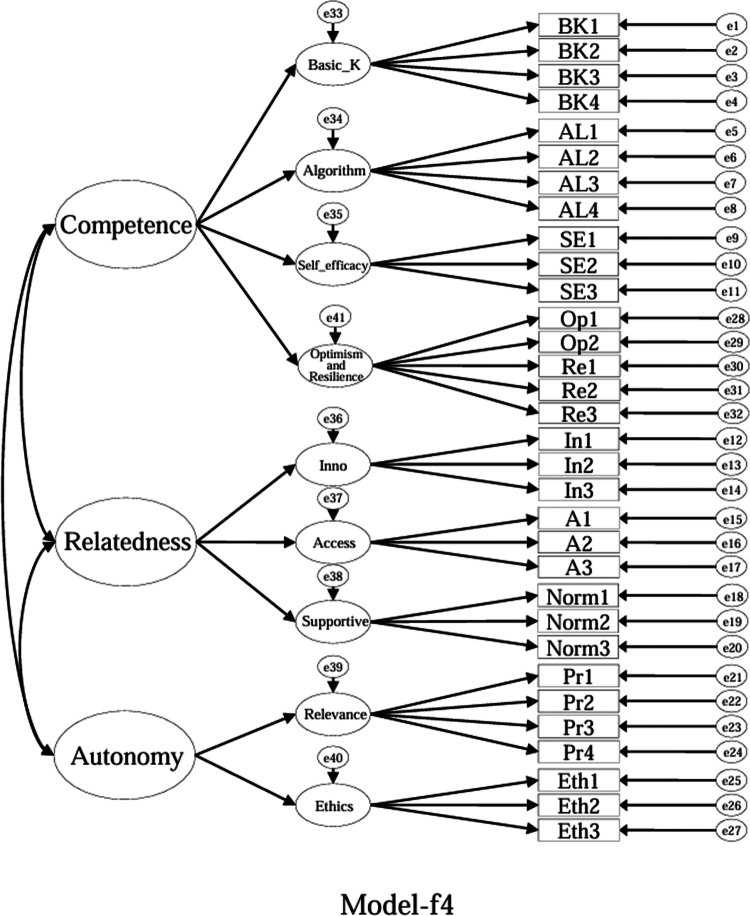
**Model-f4**: SDT-aligned second-order model with *Ethics* specified under *Autonomy* and ‘*Optimism & Resilience*’ specified under *Competence* (admissible solution).

**Figure 10.**
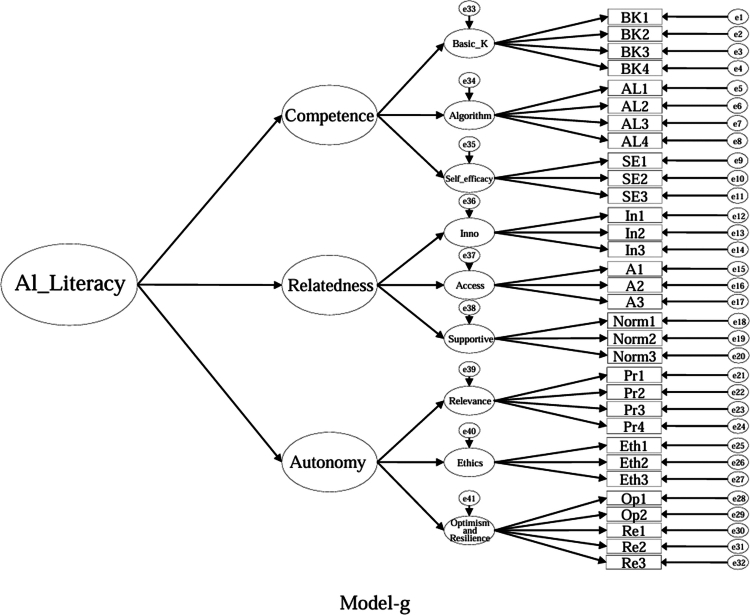
**Model-g**: Third-order general-factor model (inadmissible solution).

Mapping the ten candidate facets onto SDT yields three domains populated by ten hypothesised facets: *Competence* (*Basic knowledge, Algorithms, and Self-efficacy in AI learning), Relatedness (Innovative design for medical benefits, Access to support and technology, and Supportive social norms), and Autonomy (Personal relevance, Ethics, Optimism, and Resilience*). Because these facets were specified from prior work and then mapped onto SDT, the SDT-aligned higher-order CFA models are interpreted as tests of theoretical overlap and parsimony. This mapping clarifies the theoretical organisation of the facets and guides the prespecification of SDT-aligned higher-order CFA models for comparison. Guided by SDT, multiple higher-order organisations are theoretically defensible, particularly the alternative placement of *Ethics* under *Competence*, *Relatedness*, or *Autonomy*. Anticipating this, we prespecified a family of CFA models (**Models a–g**; Figures 1–10) that implement these alternatives, including a correlated nine-factor solution and SDT-aligned higher-order variants. The following section outlines the model specifications and evaluation plan, and the subsequent Results section presents the comparative fit (Table 7).

## Methods

### Participants

This study was conducted at a medical college in northern Taiwan. A total of 880 undergraduate medical students (Years 1−6) were invited to participate. We received 549 questionnaires (response rate = 62.4%). We excluded 31 questionnaires with missing responses to one or more ALSMS items [41,42], yielding 518 complete cases for analysis (analytic sample rate = 58.9%; completion rate among respondents = 94.4%). In Taiwan, undergraduate medical education typically follows a six-year undergraduate-entry structure; Years 1−4 generally emphasise preclinical/basic sciences and early clinical exposure, whereas Years 5−6 largely comprise full-time clinical clerkships. We recruited 518 undergraduate medical students (151 female, 29.2%; 367 males, 70.8%) across Years 1–6 (Year 1 = 131 [25.3%], Year 2 = 82 [15.8%], Year 3 = 77 [14.9%], Year 4 = 69 [13.3%], Year 5 = 88 [17.0%], Year 6 = 71 [13.7%]; Table 1).

**Table 1.** Demographic information of the participants in this research (*N* = 518).

Participants	*N* (%)
**Gender**	
Female	151 (29.2)
Male	367 (70.8)
**Grade, years**	
First	131 (25.3)
Second	82 (15.8)
Third	77 (14.9)
Fourth	69 (13.3)
Fifth	88 (17)
Sixth	71 (13.7)

All participants had prior exposure to institutionally offered AI-related coursework, including an introductory module within the Medical Science and Technology Programme. The study was approved by the Institutional Review Board of Tri-Service General Hospital (IRB No. B202305022). Participation was voluntary; informed consent was obtained; and questionnaires were anonymous and unlinked to course grades.

For clarity, Table 2 summarises the scale development and validation procedure used in this study. It provides a concise overview of the major methodological steps, including candidate facet development, expert review, pilot testing, participant recruitment and screening, data screening, split-sample validation, factorability assessment, EFA, CFA, and theory-driven model comparisons. The following subsections describe each step in detail.

**Table 2.** Overview of the scale development and validation procedure.

Step	Procedure	Purpose
1	Candidate facets were derived from prior AI-literacy instruments, reviews in higher education and medical contexts, and an existing AI learning intention framework.	To establish the initial construct space for medical AI literacy.
2	Ten candidate facets were adapted and contextualised for undergraduate medical education.	To ensure that the scale reflected the clinical, ethical, educational, and learner-related demands faced by medical students.
3	An interdisciplinary expert panel reviewed the draft item pool.	To examine content coverage, item-to-facet alignment, clarity, redundancy, and potential ambiguity.
4	A small pilot test was conducted with undergraduate students.	To check item comprehension and refine wording before full administration.
5	Undergraduate medical students were invited to complete the questionnaire, and incomplete ALSMS responses were excluded.	To obtain a complete analytic dataset for scale validation.
6	Questionnaires were screened for incomplete ALSMS item responses before analysis, and the analytic dataset was screened for univariate and multivariate outliers.	To verify data quality before factor analyses while retaining plausible response patterns.
7	A split-sample validation design was used, with 204 participants assigned to the EFA and 314 to the CFA.	To refine the structure in one subsample and cross-validate it in an independent subsample.
8	Factorability was evaluated using the Kaiser-Meyer-Olkin measure and Bartlett's test of sphericity before EFA.	To determine whether the data were suitable for factor analysis.
9	EFA was conducted using principal components extraction with varimax rotation.	To examine dimensionality, refine the first-order structure, and remove weak or salient cross-loading indicators.
10	CFA was conducted to cross-validate the retained first-order structure.	To evaluate factorial validity, reliability, convergent validity, discriminant validity, and model fit.
11	Prespecified model comparisons were conducted across first-order, higher-order, SDT-aligned, and sensitivity specifications.	To examine whether SDT-aligned higher-order models provided a parsimonious representation of the retained facets.
12	Inadmissible models were reported for transparency, whereas admissible models were retained for interpretation, including cautious interpretation of the sensitivity model.	To support transparent reporting and appropriate interpretation of the final measurement structure.

Note: EFA = exploratory factor analysis; CFA = confirmatory factor analysis; SDT = Self-Determination Theory; ALSMS = AI Literacy Scale for Medical Students.

### Split-sample design and rationale

From the full sample (*N* = 518), we implemented a prespecified split-sample validation (by gender and year level; reproducible seed): exploratory factor analysis (EFA) on approximately 40% (*n* = 204) to derive the initial structure and confirmatory factor analysis (CFA) on approximately 60% (*n* = 314) to cross-validate the dimensionality, consistent with guidance to conduct EFA followed by CFA on an independent sample [43]. We allocated more cases to the CFA set because the confirmatory phase entailed comparisons among nine alternative specifications, including higher-order models, for which larger samples improve solution propriety, parameter-estimate stability, and fit-index precision [44]. For the EFA, the sample size of approximately 200 participants met commonly cited adequacy guidelines (≥5–10 participants per indicator, with 32 items, resulting in approximately 6.4 participants/item) [45]. In addition, the EFA set exhibited moderate-to-high communalities (item loadings of .57–.89), further supporting adequacy at this sample size [46,47]. For the CFA, we targeted a sample size above 300, which is generally considered adequate for complex SEM models involving a large number of free parameters [48,49], and this allocation also aligns with simulation evidence showing that larger samples are required as model complexity and the number of estimated parameters increase [44].

### Factors and item development

Guided by prior AI-literacy instruments and reviews in higher-education and medical contexts [3,17,18,22], we adapted candidate facets and mapped them to SDT’s three needs: *Competence*, *Relatedness*, and *Autonomy* [11]. Specifically, we drew on an AI learning intention framework developed in our prior work, which comprises four domains spanning epistemic capacity (e.g., basic AI knowledge), practical application and resources, subjective beliefs, and psychological attitudes (e.g., optimism, resilience, personal significance) [3,40]. We then adapted and contextualised this framework for application within medical education [3].

The ten candidate facets were selected because they jointly covered three complementary requirements for medical AI literacy identified in prior AI-literacy research and in medical education contexts. First, *Basic knowledge*, *Algorithms*, and *Self-efficacy in AI learning* were retained to capture students’ conceptual understanding, procedural awareness, and perceived capacity to learn with medical AI. Second, *Innovative design for medical benefits*, *Access to support and technology*, and *Supportive social norms* were retained to capture the patient-oriented, contextual, and social supports that shape responsible AI learning. Third, *Ethics*, *Personal relevance*, *Optimism*, and *Resilience* were retained to capture value-based judgement, internalisation, and adaptive engagement with AI-related change. These facets were retained when supported by prior literature relevant to medical learners and judged by the expert panel to provide distinct, nonredundant coverage of the intended construct.

We convened an interdisciplinary expert panel of faculty (*n* = 6) with expertise in medical education, curriculum design, and educational assessment to review the draft item pool iteratively [43,50]. The panel comprised three medical-education scholars (two with >20 years of experience and one with 16 years), two curriculum-design experts (18 and 10 years of experience), and one educational assessment expert (25 years of experience). In these meetings, reviewers examined (i) content coverage of the intended facets, (ii) alignment between each item and its target facet, (iii) clarity/readability for undergraduate medical students, and (iv) redundancy and potential ambiguity. Revisions were made through group consensus. We then conducted a small pilot with 10 undergraduate students to assess item comprehension and wording; feedback led to minor refinements prior to full administration.

The resulting working pool comprised ten facets: *Basic knowledge, Algorithms, Self-efficacy in AI learning, Innovative design for medical benefits, Access to support and technology, Supportive social norms, Ethics, Personal relevance, Optimism,* and *Resilience*.

To make the SDT-based mapping more transparent, we further specified the rationale for assigning each candidate facet to its respective SDT domain. Facets were assigned to *Competence* when their item content reflected knowledge, procedural understanding, or perceived effectiveness in learning with medical AI; to *Relatedness* when their item content reflected social, institutional, contextual, or patient-oriented supports that connect learners to others and shared clinical goals; and to *Autonomy* when their item content reflected personally endorsed value, ethical responsibility, or self-directed persistence in engaging with medical AI. Table 3 summarises the rationale for each facet-to-domain assignment.

**Table 3.** Rationale for assigning ALSMS candidate facets to SDT domains.

Candidate facet	Assigned SDT domain	Rationale for assignment
Basic knowledge	Competence	This facet reflects students’ conceptual and technical understanding of how AI functions in medical contexts, including medical image analysis, voice consultation systems, intelligent devices, and human-computer interaction. It was assigned to *Competence* because such understanding supports learners’ perceived capacity to engage effectively with medical AI.
Algorithms		This facet reflects procedural and logical understanding of algorithmic operations, including conditional expressions, programme outcomes, and error identification. It was assigned to *Competence* because these skills support students’ ability to interpret, evaluate, and troubleshoot AI-related processes.
Self-efficacy in AI learning		This facet captures students’ confidence in learning medical AI concepts and succeeding in AI-related coursework. It was assigned to *Competence* because perceived effectiveness and confidence in mastering learning tasks are central to the competence need in SDT.
Innovative design for medical benefits	Relatedness	This facet reflects students’ orientation toward improving medical AI applications for patient benefit, patient populations, and patient care. It was assigned to *Relatedness* because the item content links AI learning to the welfare of others and to shared clinical goals, rather than to individual technical skill alone.
Access to support and technology		This facet reflects the availability of information, open resources, and help when students need to learn more about medical AI. It was assigned to *Relatedness* because these forms of social and contextual support create a connected learning environment that enables participation in AI-related learning.
Supportive social norms		This facet reflects encouragement and endorsement from parents, teachers, mentors, and classmates. It was assigned to *Relatedness* because these social signals indicate that AI learning is valued by important others and by the surrounding educational community.
Ethics	Autonomy	This facet reflects students’ commitment to ethical guidelines and principles, and to avoiding AI use that may lead to unethical outcomes in clinical or medical research contexts. It was assigned to *Autonomy* because the item content emphasises value-congruent judgement and self-endorsed responsibility in deciding how AI should be used in care and research.
Personal relevance		This facet reflects whether students perceive AI technologies as meaningful for their own clinical learning, including task completion, performance, productivity, and efficiency. It was assigned to *Autonomy* because perceived personal relevance supports internalisation and self-endorsed engagement with AI learning.
Optimism		This facet reflects a positive expectancy toward medical AI and willingness to engage constructively with AI-related change under uncertainty. It was assigned to *Autonomy* because the item content emphasises self-directed orientation toward future learning and adaptation.
Resilience		This facet reflects adaptive persistence and constructive self-development in response to the growing role of medical AI. It was assigned to *Autonomy* because the item content emphasises sustained, self-endorsed engagement and continued development in response to AI-related change. Its possible relation to *Competence* was also acknowledged and examined through the sensitivity model.

To aid interpretability, we provide one illustrative item per facet (the selected item from Appendix A): *Basic knowledge*: ‘I understand how computers process images for intelligent medical image analysis’; *Algorithms*: ‘I can understand conditional expressions (e.g., if-else)’; *Self-efficacy in AI learning*: ‘I am confident that I will achieve good grades in courses related to medical AI’; *Innovative design for medical benefits*: ‘I enjoy thinking about how to improve medical AI applications to better serve patients’; *Access to support and technology*: ‘I can easily access information about medical AI’; *Supportive social norms*: ‘My teachers (or mentors) emphasise the importance of creative learning through medical AI technologies’; *Ethics*: ‘In clinical or medical research, I would never use AI technologies that may lead to unethical outcomes’; *Personal relevance*: ‘Using AI technologies helps me complete clinical learning tasks more quickly’; *Optimism*: ‘In the field of medical AI, even in uncertain situations, I expect the best outcomes’; *Resilience*: ‘When I realise that medical AI is becoming increasingly powerful, I consider how to develop myself further.’ All items were rated on a 5-point Likert scale, ranging from 1 (strongly disagree) to 5 (strongly agree). Guided by the SDT-based mapping described in the Introduction, we generated items for the ten factors (Appendix A). We examined their internal structure using an EFA followed by a CFA on independent subsamples. Consistent with this facets-first logic, SDT was used to organise candidate facets into a priori theoretical structure, which was subsequently evaluated through EFA, CFA, and model comparisons.

### Data analysis

We screened the analytic dataset at the case level prior to factor analyses. Missing data were handled via complete-case analysis: questionnaires with incomplete ALSMS item responses were excluded prior to analysis, and the analytic dataset contained no missing ALSMS values [41,42]. We screened univariate outliers using standardised facet composite scores (|z| > 3.29) [51] and screened multivariate outliers using Mahalanobis distance [52] computed from the nine facet composite scores (χ² with df = 9, *p* < .001). In the CFA subsample (*n* = 314), this procedure flagged 2 potential univariate outliers and 13 potential multivariate outliers. All flagged cases were inspected and retained because responses fell within the valid Likert range and showed no evidence of data-entry errors or implausible response patterns [51,53].

Factor analyses followed a prespecified split-sample design, as described earlier (EFA set: approximately 40%, CFA set: approximately 60%). We first conducted an EFA in IBM SPSS Statistics (Statistical Package for the Social Sciences; SPSS, version 25.0; IBM Corp., Armonk, NY) using a principal components extraction with varimax rotation to examine dimensionality and screen items; indicators with primary loadings <.50 or salient cross-loadings were removed. Prior to EFA, we evaluated factorability using the Kaiser–Meyer–Olkin (KMO) measure [54] and Bartlett’s test of sphericity [55]. Although the candidate facets were assembled a priori from prior instruments, EFA served as a data-driven refinement step to evaluate dimensionality, remove weak or cross-loading indicators, and empirically consolidate closely related facets when supported by the data [46].

The EFA-retained number of first-order dimensions (k) determined the number of latent variables for subsequent confirmatory analyses. We performed CFA in IBM SPSS Amos (Analysis of Moment Structures; AMOS, version 21.0; IBM Corp., Armonk, NY) to cross-validate the retained first-order structure and to test theoretically motivated higher-order representations aligned with SDT. Model fit was evaluated using the chi-square/degrees of freedom ratio (χ²/df), root mean square error of approximation (RMSEA), standardised root mean square residual (SRMR), goodness-of-fit index (GFI), adjusted goodness-of-fit index (AGFI), comparative fit index (CFI), incremental fit index (IFI), and Tucker–Lewis index (TLI), interpreted against conventional guidelines. In brief, RMSEA and SRMR summarise approximate and residual-based misfit (lower values indicate better fit), whereas GFI/AGFI and CFI/IFI/TLI provide absolute and incremental indices of overall model fit (higher values indicate better fit). Evidence for reliability and validity included Cronbach’s *α*, composite reliability (CR), and average variance extracted (AVE); discriminant validity was examined using the Fornell-Larcker criterion [56]. Finally, we compared nine theoretically motivated model specifications (first-order and hierarchical variants) plus one SDT-aligned sensitivity specification (Model-f4) to evaluate an alternative placement of the ‘*Optimism & Resilience*’ facet; inadmissible solutions were reported for completeness but excluded from comparative interpretation.

### Model specifications and comparison plan (CFA)

Using the EFA-retained first-order structure (k = 9 facets) as the measurement layer and SDT as the organising framework, we prespecified a set of CFA models for theory-driven comparison (see Figures 1-10). The candidate models included: (a) an independence (null) model; (b) a first-order one-factor model; (c) a first-order k-factor uncorrelated model; (d) a first-order k-factor correlated model; (e) a second-order general-factor model in which the k first-order facets load on a single higher-order AI-literacy factor; (**f1–f3**) three SDT-aligned second-order variants in which the 9 first-order facets are grouped under competence, relatedness, and autonomy, differing only in the placement of the *Ethics* facet (under *Relatedness*, *Competence*, or *Autonomy*); (**f4**) an SDT-aligned sensitivity variant of **Model-f3** in which ‘*Optimism & Resilience*’ loads on *Competence* rather than *Autonomy* (with *Ethics* remaining under *Autonomy*); and (g) a third-order general-factor model in which the three SDT domains serve as second-order factors that load on a higher-order AI-literacy factor. Model‑fit indices and decision thresholds are specified in Data Analysis. Following recommendations for construct validation using multiple theory-informed model specifications, we compared all admissible models and documented, but excluded, inadmissible solutions (e.g., non-positive definite matrices, factor correlations > 1.0, Heywood cases) from substantive interpretation.

## Results

### Exploratory factor analysis for the ALSMS

Factorability was supported across the three domain-specific EFAs (*Competence*, *Relatedness*, and *Autonomy*), with KMO values [54] ranging from 0.89 to 0.90 and Bartlett’s tests of sphericity [55] significant in all cases (all *p* < .001). Using the EFA subsample (*n* = 204; see Methods), a principal-components solution with varimax rotation supported a nine-factor structure. Within the set of autonomy-related items, ‘*Optimism*’ and ‘*Resilience*’ converged into a single factor. For interpretability, we refer to this combined facet as ‘*Optimism & Resilience*’ and discuss it in relation to our SDT mapping in the subsequent model comparisons. The remaining facets aligned with the a priori mapping. Standardised loadings ranged from .57 to .89, and the Cronbach’s *α* for the retained factors ranged from .81 to .94, explaining between 11.71% and 55.53% of the relevant variance (Table 4). For interpretability, the nine first-order factors are organised under the three SDT domains: *Competence*, *Relatedness*, and *Autonomy*. These findings thus confirmed the reliability and robustness of the ALSMS, thus highlighting its suitability for further confirmatory factor analysis.

**Table 4.** EFA of the AI literacy scale for medical students (ALSMS) (*N* = 204).

Dimension/factor	Items, n	Mean ± SD	Cronbach’s alpha	Factor loading (Min–Max)	Variance explained (%)
**Competence**					
Basic knowledge	4	3.14 ± 0.86	0.90	0.83–0.85	15.61
Algorithms	4	3.66 ± 0.69	0.91	0.77–0.87	54.60
Self-efficacy in AI learning	3	3.54 ± 0.75	0.88	0.81–0.84	9.40
**Relatedness**					
Innovative design for medical benefits	3	3.62 ± 0.74	0.86	0.59–0.85	10.41
Access to support and technology	3	3.51 ± 0.77	0.91	0.81–0.87	62.24
Supportive social norms	3	3.70 ± 0.70	0.81	0.57–0.84	7.28
**Autonomy**					
Ethics	3	3.88 ± 0.70	0.85	0.76–0.89	11.71
Personal relevance	4	3.73 ± 0.75	0.93	0.83–0.87	14.20
Optimism and Resilience	5	3.82 ± 0.70	0.94	0.78–0.87	55.53

### Confirmatory factor analysis for the ALSMS

A confirmatory factor analysis (CFA) was conducted with 314 participants to assess the construct validity of the AI Literacy Scale for Medical Students (ALSMS). The CFA results, summarised in Table 5, demonstrated strong factor loadings for all items (ranging from 0.62 to 0.94), exceeding the recommended threshold of 0.50 and achieving statistical significance (*p* < 0.001) [57]. The analysis validated a nine-factor structure that aligned with the three dimensions of Self-Determination Theory (SDT). The average variance extracted (AVE) values were greater than 0.5, confirming that the factors explained a significant proportion of variance. Additionally, composite reliability (CR) values ranged from 0.77 to 0.93, surpassing the 0.60 threshold, thereby establishing convergent construct validity [58]. The Cronbach's alpha coefficients for the individual factors ranged from 0.77 to 0.94, and the overall Cronbach’s alpha for the scale was 0.96, indicating excellent internal consistency. In addition, the model fit indices for this CFA model were: χ^2^/df = 2.26, RMSEA = 0.064, SRMR = 0.045, GFI = 0.84, AGFI = 0.81, CFI = 0.93, IFI = 0.93, and TLI = 0.92 [49,57,59]. We note that GFI/AGFI are marginal, whereas RMSEA/SRMR/CFI/IFI/TLI/indicate good fit. These results collectively confirm the robustness and reliability of the ALSMS, thus supporting its use as an appropriate measure of AI literacy among medical students.

**Table 5.** CFA of the AI literacy scale for medical students (ALSMS) (*N* = 314).

Factor and item	Factor loading	t value	AVE	CR	Cronbach’s alpha	Mean ± SD
**Basic knowledge (BK)**	–	–	0.66	0.89	0.89	3.04 ± 0.84
BK1	0.80	–				
BK2	0.87	17.08^*^				
BK3	0.77	14.73*				
BK4	0.81	15.71*				
**Algorithms (AL)**	–	–	0.67	0.89	0.89	3.67 ± 0.64
AL1	0.83	–				
AL2	0.76	15.07*				
AL3	0.86	18.03*				
AL4	0.83	17.10*				
**Self-efficacy in AI learning (SE)**	–	–	0.68	0.86	0.86	3.53 ± 0.72
SE1	0.76	–				
SE2	0.85	15.15*				
SE3	0.87	15.42*				
**Innovative design for medical benefits (In)**	–	–	0.63	0.84	0.84	3.53 ± 0.75
In1	0.79	–				
In2	0.77	14.06*				
In3	0.82	15.1*				
**Access to support and technology (A)**	–	–	0.67	0.86	0.86	3.51 ± 0.69
A1	0.79	–				
A2	0.87	16.59*				
A3	0.80	14.97*				
**Supportive social norms (Norm)**	–	–	0.53	0.77	0.77	3.61 ± 0.66
Norm1	0.67	–				
Norm2	0.76	11.59*				
Norm3	0.75	11.49*				
**Ethics (Eth)**	–	–	0.72	0.88	0.94	3.87 ± 0.82
Eth1	0.94	–				
Eth2	0.94	25.90*				
Eth3	0.62	12.82*				
**Personal relevance (Pr)**	–	–	0.78	0.93	0.93	3.73 ± 0.72
Pr1	0.84	–				
Pr2	0.90	20.90*				
Pr3	0.92	21.88*				
Pr4	0.87	19.89*				
**Optimism and Resilience**	–	–	0.66	0.91	0.91	3.81 ± 0.63
OP1	0.81	–				
OP2	0.80	16.32*				
Re1	0.80	16.30*				
Re2	0.83	17.18*				
Re3	0.82	16.81*				

Abbreviations: AVE: average variance extracted; CFA: confirmatory factor analysis; CR: composite reliability; SD: standard deviation.

Note: Total Cronbach's alpha coefficient: 0.96.

^*^ *P* < .001.

### Intercorrelations and discriminant validity of the ALSMS

Table 6 presents the intercorrelations among the nine factors.

**Table 6.** Intercorrelations among the nine factors included in the ALSMS (*N* = 314).

Variables	1	2	3	4	5	6	7	8	9
1. Basic knowledge	**(0.81)**								
2. Algorithms	0.36*	**(0.82)**							
3. Self-efficacy in AI learning	0.36*	0.58*	**(0.82)**						
4. Innovative design for medical benefits	0.57*	0.52*	0.56*	**(0.79)**					
5. Access to support and technology	0.49*	0.48*	0.54*	0.53*	**(0.82)**				
6. Supportive social norms	0.41*	0.58*	0.55*	0.62*	0.66*	**(0.73)**			
7. Ethics	0.34*	0.43*	0.40*	0.56*	0.45*	0.42*	**(0.85)**		
8. Personal relevance	0.38*	0.56*	0.60*	0.55*	0.60*	0.63*	0.52*	**(0.88)**	
9. Optimism and Resilience	0.34*	0.64*	0.68*	0.61*	0.56*	0.65*	0.52*	0.69*	**(0.81)**

Note: The square roots of the AVE values are presented on the diagonal in bold. The off-diagonal values represent the correlations among the relevant factors.

^*^ *P* < .001 (two-tailed).

The correlation coefficients among the factors in this scale indicated that all were statistically significant (*p* < 0.001), with values ranging from 0.34 to 0.69. The highest correlation in this context was between *Personal relevance*’ and ‘*Optimism and Resilience*’ (r = 0.69, *p* < 0.001), while the second-highest was between ‘Self-efficacy in AI learning’ and ‘*Optimism and Resilience*’ (r = 0.68, *p* < 0.001). The results regarding the discriminant validity of the scale, as determined by reference to the square root of the average variance extracted (AVE) for each factor, were greater than the corresponding correlations. This criterion, proposed by Fornell and Larcker [58], confirmed that each factor was distinct and measured a unique aspect of AI literacy among medical students. Hence, the measurement model's discriminant validity was confirmed.

### Comparison of the fit statistics of the proposed models

Several fit indices, including χ²/df, RMSEA, SRMR, GFI, AGFI, CFI, IFI, and TLI, were examined to conduct a thorough investigation of the proposed models for the multidimensional ALSMS among medical students. According to established guidelines [58,59], a good model fit is indicated by χ^2^/df values less than 3.0, RMSEA, and SRMR values below 0.08, and GFI, AGFI, CFI, IFI, and TLI values above 0.90 (marginal acceptance applies to GFI and AGFI values that are at least above 0.80).

As indicated in Table 7, **Model-a** (the null model, Figure 1), **Model-b** (the first-order, one-factor model, Figure 2), and **Model-c** (the first-order uncorrelated model, Figure 3) exhibited a poor fit; namely, the corresponding indices failed to reach the acceptable thresholds. In contrast, **Model-d** (the correlated model, Figure 4) exhibited the best fit among the proposed models; namely, χ^2^/df = 2.26, RMSEA = 0.064, SRMR = 0.045, GFI = 0.84, AGFI = 0.81, CFI = 0.93, IFI = 0.93, and TLI = 0.92, all of which met or exceeded the suggested values. However, although **Model-e** (Figure 5) exhibited acceptable values for most indices, it did not qualify because its AGFI (0.79) was below the acceptable threshold.

**Table 7.** Fit indices of the proposed models included in the multidimensional AI literacy instrument for medical students.

Model	χ^2^/df	RMSEA	SRMR	GFI	AGFI	CFI	IFI	TLI
Model-a	16.40	0.222	0.430	0.14	0.09	<0.01	<0.01	<0.01
Model-b	7.04	0.140	0.094	0.56	0.49	0.63	0.64	0.61
Model-c	5.57	0.121	0.384	0.55	0.49	0.72	0.72	0.70
Model-d	2.26	0.064	0.045	0.84	0.81	0.93	0.93	0.92
Model-e	2.42	0.067	0.056	0.82	0.79	0.92	0.92	0.91
Model-f1^†^	2.40	0.067	0.057	0.83	0.80	0.92	0.92	0.91
Model-f2^†^	2.42	0.067	0.057	0.82	0.79	0.92	0.92	0.91
Model-f3	2.41	0.067	0.057	0.83	0.80	0.92	0.92	0.91
Model-f4	2.40	0.067	0.058	0.82	0.80	0.92	0.92	0.91
Model-g^†^	2.44	0.067	0.057	0.83	0.80	0.92	0.92	0.91
Suggested values	<3.0	<0.08	<0.08	>0.80	>0.80	>0.90	>0.90	>0.90

Note: Models marked with ‘†’ yielded inadmissible solutions (e.g., non–positive definite covariance matrices, factor correlations > 1.0, or Heywood cases). Fit indices are reported for completeness but are not interpreted or used for model comparison. These inadmissible solutions may reflect overparameterization in the presence of high inter-factor correlations; therefore, we retain admissible, interpretable first- and second-order solutions for substantive conclusions. **Model-f4** is a sensitivity SDT-aligned variant of Model-f3 that relocates ‘Optimism and Resilience’ under Competence (‘Ethics’ remains under Autonomy).

Although the software produced fit indices for **Model-f1** (Figure 6), **Model-f2** (Figure 7), and **Model-g** (Figure 10), these solutions were inadmissible due to non–positive definite covariance matrices, factor correlations exceeding 1.0, or negative variance estimates (Heywood cases). Accordingly, these inadmissible models are reported for completeness but are neither interpreted nor included in the comparative evaluation. The admissible sensitivity model, **Model-f4** (Figure 9), was retained for cautious interpretation because it tested the alternative placement of ‘*Optimism and Resilience’* under *Competence*. In contrast, **Model-f3**, a second-order SDT-aligned model with *Ethics* specified under *Autonomy* (Figure 8), yielded an admissible solution with acceptable fit and was therefore retained for substantive interpretation. These identification problems likely reflect over-parameterisation and high intercorrelations among higher-order factors, suggesting that a third-order general factor may be empirically redundant given the strength of the first-order relations in this sample.

Specifically, with respect to the SDT structure that guided the model design, **Model-f1** assigned ‘*Ethics*’ to ‘*Relatedness*,’ **Model-f2** assigned ‘*Ethics*’ to ‘*Competence*,’ and **Model-f3** (the second-order, three-factor model) assigned ‘*Ethics*’ to ‘*Autonomy*.’ **Model-f3**, however, was the only positively defined model among these three models that exhibited acceptable fit indices (χ^2^/df = 2.41, RMSEA = 0.067, SRMR = 0.057, GFI = 0.83, AGFI = 0.80, CFI = 0.92, IFI = 0.92, and TLI = 0.91).

While ‘*Ethics*’ could plausibly load under ‘*Relatedness*’ (professional norms) or ‘*Competence*’ (governance knowledge), assigning ‘*Ethics*’ to ‘*Autonomy*’ (**Model-f3**) yielded an admissible and well-fitting structure. We treat this as one theoretically defensible organisation, rather than the only viable one. Accordingly, we report both the first-order correlated nine-factor model and the second-order SDT-aligned model for transparent use across contexts. Overall, **Model-d** (a first-order correlated nine-factor model) provided the best and most robust fit for the ALSMS, whereas **Model-f3** (a second-order, three-factor model with *Ethics* under ‘*Autonomy’*) also achieved an acceptable fit and serves as a feasible alternative.

We additionally tested **Model-f4** (Figure 9), a sensitivity SDT-aligned variant that relocates the ‘*Optimism & Resilience*’ facet under *Competence* (while keeping *Ethics* under *Autonomy*). **Model-f4** yielded an admissible solution with acceptable fit (χ²/df = 2.40, RMSEA = 0.067, SRMR = 0.058, GFI = 0.82, AGFI = 0.80, CFI = 0.92, IFI = 0.92, TLI = 0.91), comparable to **Model-f3** (Table 7). These results suggest that the higher-order placement of ‘*Optimism & Resilience*’ may be theoretically plausible under either domain and should be interpreted as a parsimonious summary rather than a unique mapping.

Furthermore, to highlight the potential to integrate all the components of AI literacy into a single structure, three distinct orders (i.e., first-, second-, and third-order) one-factor models (i.e., **Models -b**, **-e**, and **-g**) were created for this study. The findings suggest that it is not feasible to account for students' AI literacy within a single-component framework.

## Discussion

### Principal findings and contributions

This study provides validity evidence for interpreting ALSMS scores as indicators of medical students’ AI literacy within a Self-Determination Theory (SDT)-informed framework. Rather than treating AI literacy as a primarily technical, attitudinal, or readiness-based construct, the ALSMS conceptualises medical AI literacy as a multidimensional construct that includes competence-, relatedness-, and autonomy-related facets. This structure builds on broader AI literacy scholarship, which has emphasised knowledge, use, evaluation, creation, and ethical engagement with AI [15–19,23], and extends this work by organising medical AI literacy through SDT’s three basic psychological needs [11–13,25,26].

The contribution of the ALSMS is therefore not limited to the development of another AI literacy instrument. Prior work has developed general AI literacy scales [23] and SDT-based measures of motivation in AI-supported learning contexts [24], but these studies were not designed to assess AI literacy in medical education. In medical education, existing empirical work has often assessed AI readiness, attitudes toward AI, interest in AI literacy curriculum, or self-assessed AI literacy using pre-existing instruments [20–22]. By contrast, drawing on and extending prior work on AI learning intention and medical students’ behavioural intentions toward learning AI [3,27], the ALSMS provides a framework for examining how students’ knowledge, self-efficacy, access to support, social norms, perceived relevance, ethical judgement, optimism, and resilience jointly shape their engagement with medical AI. This contribution is particularly relevant in medical education, where AI literacy is inseparable from clinical responsibility, patient safety, professional accountability, and human oversight [6–10,14].

Extending SDT into AI literacy also provides a meaningful way to understand the motivational and ethical dimensions of medical AI learning. The competence domain captures students’ knowledge, algorithmic understanding, and confidence in learning with AI; the relatedness domain captures the social, institutional, and patient-oriented supports that shape participation in AI learning; and the autonomy domain captures personally meaningful, value-aligned, and ethically responsible engagement with AI [11–13,25,26]. Within this framework, ethical AI use is not merely compliance with external rules, but is part of students’ internalised professional responsibility when deciding how AI should be used in clinical learning and care [6–9,60]. In rapidly evolving AI-integrated healthcare environments, such a theory-informed instrument can help educators move beyond asking whether students are simply ‘ready’ for AI and instead examine whether they are prepared to engage with AI in technically informed, socially supported, and ethically accountable ways [7,10,14,15].

### Ethical AI literacy as internalised professional responsibility

*Ethics* occupies a central position in medical AI literacy because responsible AI use requires more than technical understanding. In the ALSMS, ethics reflects students’ value-congruent responsibility for issues such as privacy, fairness, accountability, oversight, and potential harm [6–9]. Interpreting ethics through the autonomy domain of SDT highlights the importance of self-endorsed professional judgement in AI-enabled care, while recognising that ethical AI practice is also supported by competence in AI governance and by relatedness through mentorship, institutional expectations, and professional norms [11–13,25,26,60]. This multidimensional interpretation may be especially useful for curriculum design because it encourages educators to teach AI ethics not only as regulatory knowledge, but also as a process of internalised professional responsibility.

### Optimism and resilience as adaptive engagement with AI-related change

The combined ‘*Optimism & Resilience*’ facet suggests that students’ engagement with medical AI also depends on how they respond to uncertainty and change. In AI-integrated healthcare environments, learners must not only acquire technical knowledge but also sustain adaptive engagement as tools, workflows, and ethical expectations evolve [10,14]. This facet may function as a cross-domain psychological resource, connecting autonomy-related self-direction with competence-related confidence and persistence [12,38,40]. From an educational perspective, this finding suggests that AI literacy curricula should help students develop both confidence and adaptive capacity, rather than focusing only on immediate technical proficiency. Future studies should further examine whether this facet is best interpreted as part of autonomy, competence, or as a bridging resource across SDT domains.

### Model architecture and theoretical implications

ALSMS comprises nine first-order facets that capture multidimensional medical AI literacy; SDT provides a theory-informed organising lens to summarise these facets into competence, relatedness, and autonomy profiles for interpretation and educational application. Evidence for a robust first-order structure supports the multidimensionality of AI literacy, indicating that it extends beyond a single global trait. The admissible second-order solution (*Competence*, *Relatedness*, *Autonomy*) offers a parsimonious representation for theory-aligned profiling and the design of need-supportive educational interventions [12,61]. Inter-factor correlations were moderate to high (up to r = .69 between ‘*Personal relevance*’ and ‘*Optimism & Resilience*’), justifying the use of a higher-order representation for need-aligned profiling while retaining diagnostically useful first-order facets.

Conceptually, this integration allows AI literacy facets (including ethical value alignment and social-contextual supports) to be interpreted through a motivational architecture that is actionable for curriculum design and evaluation.

### Implications for medical education

A theory-driven literacy instrument allows alignment of curriculum, teaching, and assessment with SDT by translating the three needs into actionable instructional moves [12,38,61]:

***Autonomy*** (endorsement rather than independence). Autonomy-supportive AI literacy teaching can preserve the necessary clinical structure while increasing students’ sense of volition and value alignment [13,26]. In practice, educators can: (i) invite learners’ perspectives about AI use (e.g., perceived benefits, concerns, and uncertainty), (ii) acknowledge and normalise emotions that accompany AI adoption (e.g., excitement, frustration, or anxiety), (iii) provide explanatory rationales for when and why AI use should be encouraged or restricted (including governance and patient-safety considerations), (iv) offer meaningful choices within clear boundaries (e.g., selecting among case topics, tools, or roles in an AI-supported learning task), and (v) use informational, non-controlling language when communicating expectations and policies [6,12,26]. Ethics and values can be strengthened through facilitated discussion and structured reflection tasks that require students to articulate and justify value-based positions (e.g., fairness, privacy, accountability, and oversight) in realistic clinical scenarios [6–9,61].

***Competence***. *Competence* support can be operationalized through structure, scaffolding, and mastery-oriented feedback that help students experience effectiveness when learning with and about AI [12,26,61]. Educators can sequence learning from foundational concepts (how models work; limitations and bias) to increasingly complex applications (AI-assisted clinical reasoning), provide worked examples and supervised practice, and incorporate low-stakes ‘sandbox’ activities where learners can safely experiment, make errors, and receive timely, specific formative feedback [1,4,22,62]. Case-based simulations and iterative feedback can strengthen both technical understanding and self-efficacy for engaging with AI-enabled clinical workflows [26,62].

***Relatedness***. *Relatedness* support can be strengthened by creating a learning climate of belonging and reliable interpersonal support, especially for learners who may feel isolated or intimidated by rapidly evolving AI technologies [12,61,63]. Practical approaches include near-peer coaching, faculty mentorship, and interdisciplinary team projects that pair medical students with data-science/engineering collaborators around shared clinical goals [1,10,28]. Establishing peer learning communities and visible normative endorsement of responsible AI practice (e.g., through role modelling, shared standards, and supportive feedback cultures) can further reinforce connectedness and sustained engagement [7,10,26].

Beyond design guidance, ALSMS can support programme evaluation (pre–post changes in targeted facets), advising (individual need profiles), and quality improvement (mapping course features onto autonomy-, competence-, and relatedness-supportive practices to identify gaps and refine AI-literacy curricula over time) [10,25].

Although ALSMS was developed for undergraduate medical students, the SDT-anchored facet structure may also be useful for profiling AI literacy among postgraduate trainees and practicing healthcare professionals who increasingly interact with AI-enabled clinical workflows [1]. With minor contextual adaptations (e.g., referencing clinical decision support systems, documentation assistants, or institutional AI governance processes), ALSMS could support continuing professional development, workforce needs assessment, and evaluation of AI implementation training in hospitals [9]. However, such applications require additional validation, including multisite replication and measurement invariance testing across professional roles and practice settings [49].

### Research implications

Future studies can treat AI literacy as a predictor (e.g., of performance in AI-augmented OSCE stations, clinical reasoning quality, or longitudinal engagement) or as an outcome (e.g., response to targeted instructional interventions) [3,19]. To strengthen validity arguments, we recommend: (i) adding criterion-related and predictive evidence (course grades, performance-based AI tasks), (ii) testing measurement invariance across gender and training levels, (iii) multisite replication to evaluate generalisability, and (iv) longitudinal trajectories to assess whether motivational/ethical profiles predict sustained engagement and competence development. Multisite studies across medical schools (and potentially other health-professions programmes) will be particularly valuable to test the stability of the factor structure and the SDT-aligned higher-order mappings across institutional contexts [50]. Beyond institutional replication, future work should adapt and validate ALSMS for postgraduate trainees, practicing clinicians (and other health-professional groups), and examine measurement invariance across roles (student, resident, or clinician) and workplace contexts [10,49].

### Strengths and limitations

Strengths include a split-sample EFA/CFA design, comparison of theoretically motivated higher-order variants, and strong internal consistency with convergent and discriminant validity. The split-sample EFA and CFA design strengthens internal-structure validity by pairing empirical dimensionality refinement with independent confirmatory testing, supporting the stability and curricular interpretability of the competency framework. Additional strengths include the large sample size relative to the number of instrument items, the explicit mapping of factors onto Self-Determination Theory (SDT), and the transparent reporting of inadmissible models, which enhances replicability. Although our data were collected from a single institution, the split-sample EFA and CFA cross-validation and theory-driven model-comparison strategy strengthen confidence in the reproducibility of the internal structure. Nevertheless, external validity should be established through multisite replication and measurement-invariance testing across institutions and curricular contexts.

Limitations include single-site sampling, potential self-selection bias, and reliance on self-report data. Because the validity evidence for the ALSMS was derived from undergraduate medical students at a single medical college in Taiwan, the findings should be interpreted within the cultural, institutional, and curricular context of Taiwanese medical education. Students’ perceptions of AI literacy, ethical responsibility, autonomy, social norms, and support for AI learning may be shaped by local educational structures, healthcare systems, institutional AI learning opportunities, and culturally embedded expectations regarding teacher authority, peer influence, and technology adoption. Therefore, the present findings should not be generalised directly to medical students in other countries, healthcare systems, or professional training pathways. Future studies should conduct cross-cultural validation, multisite replication, and measurement invariance testing across countries, institutions, gender groups, training levels, and professional roles. Our internal structure evidence should also be complemented by behavioural, observational, and criterion-related evidence, such as AI-based performance tasks, clinical reasoning assessments, course outcomes, or longitudinal indicators of ethical and responsible AI engagement. Finally, because **Model-f3** and **Model-f4** yielded comparable fit in this sample, the higher-order placement of ‘*Optimism & Resilience*’ should be interpreted cautiously and verified in culturally and institutionally diverse samples.

## Conclusion

Anchored in SDT, the ALSMS offers a reliable, theory-informed measure of medical students’ AI literacy and clarifies how motivational and ethical processes organise this construct. A correlated nine-factor solution best captured its multidimensional nature. By explicitly contrasting correlated and SDT-aligned hierarchical specifications with alternative models (e.g., null, unidimensional, uncorrelated, and higher-order general-factor variants), our approach strengthens the construct-validation argument, demonstrates that unidimensional representations are inadequate, and supports transparent scoring decisions using either facet-level diagnostic scores or SDT-aligned domain profiles.

In contrast, admissible SDT-aligned second-order solutions provide parsimonious profile-level summaries: **Model-f3** places ‘*Ethics*’ under *Autonomy*, and a sensitivity variant (**Model-f4**) places ‘*Optimism & Resilience*’ under *Competence* with comparable fit (Table 7). These findings suggest that the higher-order placement of ‘*Optimism & Resilience*’ may vary across operationalizations and contexts, while the correlated nine-factor model remains the primary representation of ALSMS. The instrument can inform the need for a supportive curriculum design and programme evaluation of AI initiatives. To consolidate generalisability, future multi-site studies should test measurement invariance across gender, training levels, and professional roles, incorporate behavioural/performance indicators, and examine longitudinal trajectories of competence development and ethical engagement. Together, these steps would establish a scalable evidence base for AI-literacy education that integrates ethical accountability with technical competence in medicine.

## Data Availability

The data that support the findings of this study are available from the corresponding author upon reasonable request.

## Appendix A

**Basic knowledge**

1. I understand how computers process images for intelligent medical image analysis.
2. I understand how deep learning–based AI technologies perform speech recognition tasks in online intelligent voice consultations.
3. I understand how intelligent devices use AI technologies to optimise innovative health management solutions.
4. I understand how human–computer interaction is handled in intelligent voice assistant–based online consultation systems.

**Algorithms**

1. I can understand conditional expressions (e.g., if-else).
2. I can predict the outcome of a programme that contains logical conditions.
3. I can predict the execution results when input values are given to a programme.
4. When an error occurs in programme execution, I can identify the cause.

**Self-efficacy in AI learning**

1. I am confident that I will achieve good grades in courses related to medical AI.
2. I am confident in learning the basic concepts of medical AI courses.
3. I believe that as long as I work hard in medical AI–related courses, I can succeed.

**Innovative design for medical benefits**

1. I enjoy thinking about how to improve medical AI applications to better serve patients.
2. I can envision how medical AI can be used to help patient populations.
3. I will do my best to contribute to the design of medical AI for patient care.

**Access to support and technology**

1. I can easily access information about medical AI.
2. I can continually improve my medical AI knowledge through many open-source resources.
3. When I need to know more about medical AI technologies, I can easily find help.

**Supportive social norms**

1. My parents encourage me to participate in innovative learning activities related to medical AI.
2. My teachers (or mentors) emphasise the importance of creative learning through medical AI technologies.
3. My classmates believe it is necessary to learn how to use technologies related to medical AI.

**Ethics**

1. In clinical or medical research, I would never use AI technologies that may lead to unethical outcomes.
2. In clinical or medical research, I can follow ethical guidelines when using AI technologies.
3. In clinical or medical research, I can act according to ethical principles when applying AI technologies.

**Personal relevance**

1. Using AI technologies helps me complete clinical learning tasks more quickly.
2. Using AI technologies improves my performance in clinical learning.
3. Using AI technologies increases my productivity in clinical learning.
4. Using AI technologies enhances my efficiency in clinical learning.

**Optimism and Resilience**

1. I always view the emerging field of medical AI from a positive perspective.
2. In the field of medical AI, even in uncertain situations, I expect the best outcomes.
3. When I realise that medical AI is becoming increasingly powerful, I consider how to develop myself further.
4. I build better peer and doctor–patient relationships in an increasingly intelligent world.
5. In response to the spread of AI in the medical field, I will focus on improving patient care.
